# Effects of cooking and fermentation on the chemical composition, functional, and antinutritional properties of kariya (*Hildergardia barteri*) seeds

**DOI:** 10.1002/fsn3.501

**Published:** 2017-07-25

**Authors:** Olumide S. Fawale, Saka O. Gbadamosi, Meshach M. Ige, Oseni Kadiri

**Affiliations:** ^1^ Nigerian Institute for Oceanography and Marine Research Lagos Nigeria; ^2^ Department of Food Science and Technology Obafemi Awolowo University Ile‐Ife Osun State Nigeria

**Keywords:** cooking, fermentation, functional properties, kariya, protein seed

## Abstract

The effects of natural fermentation and cooking on kariya seeds functional properties, chemical composition, and antinutritional properties were evaluated. Result showed a reduction in antinutritional properties and improvement in protein content which were observed to increase with cooking (at 100°C) and fermentation period (24–96 hr). Functional analyses showed an increase in foaming and emulsion properties, while water absorption capacity and swelling power were observed to likewise increase with an increasing temperature between 60°C and 90°C. There was also an improvement in foaming properties with increase in salt (NaCl) concentration, while emulsifying property decreases with an increase in salt (NaCl) concentration. Based on the result of the findings of this study, it can be stated that the cooking and fermentation processes employed in this study can enhance the domestic and industrial utilization of these seeds.

## 1 INTRODUCTION

Quite a number of oil seeds have been characterized but the vast majorities have not been evaluated adequately (Oderinde & Ajayi, 1998). This is also mostly valid for vegetation and crops cultivated in Nigeria which has one of the most widespread floras in Africa (Oderinde & Ajayi, 1998). In the last decade, there has been increased interest in research focusing on underutilized oil seeds for possible food product development and use (Obasi & Okolie, 1993). In Nigeria and other states in Africa, there has been increased utilization of these underutilized seeds by locals for domestic and industrial purposes. *Kariya* (*Hildegardia barteri)* falls into this group of underutilized species of plants.

*Kariya* (*Hildegardia barteri)* is predominantly an ornamental tree in West Africa that grows in Nigeria and other countries in Africa. It is called Krobo Christmas tree (Irvine, 1961), grown only for its bright beautiful flowers which bud during the dry season. The flowers of this plant which develop into one‐seeded pods are borne on leafless branches around the months of December to March annually. Each leaf of this plant is approximately 50 mm in length (Hildergadia Notes, 2009). When dried after attaining maturity, the pods drop and mostly dispose of as refuse. But in some parts of West Africa, the kernel is extracted from the pods and processed by roasting it like peanuts, with peanut flavor for human consumption (Inglett, Cavins, & Spencer, 1973). In some other instances, these kernels are used as condiments in the preparation of food by locals. In a report by Ogunsina, Olaoye, Adegbenjo, and Babawale (2011), kariya kernels were reported to contain 6.5%, 2.8%, 37.5%, and 17.5% of crude fiber, ash, crude fat, and protein. Research efforts on kariya have been limited to the physical properties of the seeds (Adebayo, Ogunsina, & Gbadamosi, 2013; Inglett et al., 1973; Ogunsina et al., 2011). Adebayo et al. (2013) have likewise made some interesting findings on the physicochemical and functional properties of Kariya flours. Gbadamosi and Famuwagun (2016) reported that the antinutrient content in Kariya seed isolates was reduced significantly when fermented. In another study by the same investigator, it was observed that when protein hydrolysate was processed from Kariya seeds and fermented, functional, antioxidant and proximate properties were improved as a result. In another related study by Adiamo, Gbadamosi, and Abiose (2015a, 2015b), the functional properties like foaming and emulsifying properties, water absorption capacity, and gelling capability were enhanced when kariya seeds were processed into its protein concentrate and isolate.

Furthermore, kariya seeds have found restricted application as food and food ingredient due to its antinutrient composition. In order to increase the utilization of underutilize seeds, Gbadamosi, Abiose, and Aluko (2012) suggested processing of the whole flour of these seeds into defatted flours and examining its suitability as functional ingredients and food supplements. It was also said that the ultimate success of utilizing any plant protein as food ingredients mainly depends on its functional and nutritional properties (Gbadamosi et al., 2012).

Fermented plant proteins are a vital nutritional source to the populace of the Oriental and African continents. Fermentation has over the years been demonstrated to improve the biological availability and enrichment of food substrates such as protein, essential amino acids, and vitamins, while also resulting in the elimination of antinutrients (McGovern et al., 2004). Fermented foods improve the digestibility, flavor, aroma, health‐promoting benefits, and availability of bio‐nutrients (Jeyaram et al., 2009). It has been reported that fermentation improves the nutritional, shelf‐life structural properties as well as reduction of antinutritional content (Fowomola & Akindahunsi, 2008). Despite the importance of these seeds, there is still a dearth of information on the functionality of kariya seed flour. This study therefore aimed to investigate the functional, chemical composition, and antinutritional properties of cooked and fermented kariya flour with a view to improving its utilization as food ingredients.

## 2 MATERIALS AND METHODS

### 2.1 Sample collection

*Kariya* (*Hildegardia barteri)* seeds for this research were harvested from various locations in University, Ile‐Ife, Nigeria. The outer shell of the seeds was manually decorticated and air‐dried in a cabinet dryer at 45°C, stored in tight and moisture‐free plastic containers for use in further analysis. All chemicals used were obtained from Sigma Chemicals, (St. Louis, MO) and were of analytical grade.

### 2.2 Preparation of fermented samples

Fermentation of *H. bateri* seeds was carried out by the method described by Fowomola and Akindahunsi (2008). The decorticated dried seeds were divided into four portions. The first part was kept as control (unfermented seeds), the second portion was cooked (100°C) but not fermented, and the third portion was fermented but not cooked. The fourth portion of the kernel was cooked and transferred into a calabash pot, lined uniformly with banana leaves (up to 5 layers) and allowed to undergo natural fermentation at ambient temperature for 5 days inside the incubator (30°C) after which the fermentation process was terminated by oven drying at 70°C. This was followed by milling to obtain flour which was afterward subjected to cold extraction process using acetone. Extraction was done on a magnetic stirrer (Cenco, Breda, Netherland) for 4 hr, while a 1:5 w/v flour‐to‐solvent ratio was adopted after which slurry gotten was filtered and residue re‐extracted as described previously. De‐solventization of the resulting flour was carried out by drying in a fume hood and ground into fine flour particles using a Kenwood grinder. Homogenous defatted flour samples were obtained using sieving through a 200‐mm sieve mesh and stored in air tight containers at 4°C until used for analysis. Likewise, some dried whole seeds were not defatted and kept under similar conditions as described previously.

### 2.3 Chemical analyses

#### 2.3.1 Proximate composition

The proximate compositions of the fermented and cooked samples were determined according to AOAC methods (2000). Ash, protein, and fat content were among factors determined. The analysis was done in triplicate while data were reported as means ± standard deviation.

### 2.4 Determination of physicochemical and functional properties

The method described by Beuchat (1977) was used for the determination of the oil absorption capacity of the samples. One gram of sample weight was centrifuged at 4,000*g* for 30 min using an MSE‐Harrier centrifuge. Swelling capacity was determined by the method described by Takashi and Seib (1998). This was carried out at room temperature and at 60°C and 90°C and expressed as milligram per gram. Water absorption capacity (WAC) was determined at room temperature and at a higher temperature between 60°C and 90°C using the technique described by Adepeju, Gbadamosi, Adeniran, and Omobuwajo (2011). Two gram of sample was used for this process and centrifugation was performed at 4,000*g* for 30 min using an MSE‐ Harrier centrifuge (Sydenham, London, UK). Water absorption was expressed as a percentage increase in the weight of the sample.

### 2.5 Protein digestibility determination

The method described by Chavan, Mckenzie, and Shahidi (2001) as modified by Gbadamosi et al. (2012) was used in the determination of protein digestibility. Sample weight of 250 mg was suspended in 15 ml of HCL of 0.1 mol/L having 1.5 mg of pepsin. This was followed by moderate shaking for a duration of 1 hr. The resultant suspension was neutralized with NaOH of 0.5 mol/L concentration and treated with 4.0 mg pancreatin in 7.5 ml of phosphate buffer (0.2 mol/L, pH 8.0). The resulting mixture was shaken at room temperature for 24 hr, filtered using What No. 1 filter paper. Residues recovered were washed with distilled water and air‐dried. This was used for protein determination using the method earlier described by AOAC (2000).

Protein digestibility was calculated using the following equation:

$$\text{In vitro protein digestibility}\left(\%\right)=\left(\frac{I-F}{I}\right)\times 100$$

where, *I* is protein content of sample before digestion and *F* is protein content of sample after digestion.

### 2.6 Antinutritional content determination

#### 2.6.1 Determination of tannins

The concentration of tannin in tested samples was determined using the modified vanillin– hydrochloric acid (MV – HCl) method of Price, Van Scoyoc, and Butler (1974).

Various concentrations (0.0, 0.1, 0.2, 0.4, 0.6, 0.8, and 1.0 mg/ml) of the catechin standard solution was pipetted into clean dried test tubes in duplicate. To one set was added 5.0 ml of freshly prepared vanillin – HCl reagent prepared by mixing equal volume of 4% (w/v) vanillin/MeOH and 16% (v/v) HCl/MeOH, and to the second set was added 5.0 ml of 4% (v/v) HCl/methanol to serve as blank. The solutions were left for 20 min before the absorbance was taken at 500 nm. The absorbance of the blank was subtracted from that of the standards. The difference was used to plot a standard graph of absorbance against concentration.

$$\text{Tannin}\left(\text{mg/g}\right)=\frac{x\text{(mg/ml)}\times 10\text{ml}}{0.2(g)}=50\text{x (mg/g)}$$

where, *x* is value obtained from standard catechin graph.

#### 2.6.2 Determination of oxalate

Oxalate was determined using titrimetric method by Falade, Dare, Bello, Osuntogun, and Adewusi (2004). Two grams of the sample was weighed in triplicate into conical flasks and extracted with a 190 ml distilled water and 10 ml 6 mol/L HCl. The suspension was placed in boiling water for 2 hr and filtered and made up to 250 ml with water in a volumetric flask. To 50 ml aliquot was added 10 ml of 6 mol/L HCl and filtered and the precipitate washed with hot water. The filtrate and the wash water was combined and titrated against conc. NH_4_OH until the salmon pink color of the methyl red indicator changed to faint yellow. The solution was heated to 90°C and 10 ml 5% (w/v) CaCl_2_ solution was added to precipitate the oxalate overnight. The precipitate was washed free of calcium with distilled water and then washed into 100 ml conical flask with 10 ml hot 25% (v/v) H_2_SO_4_ and then with 15 ml distilled water. The final solution was heated to 90°C and titrated against a standard 0.05 mol/L KMnO_4_ until a faint purple solution persisted for 30 s.

The oxalate was calculated as the sodium oxalate equivalent.

$$\begin{array}{cc} 1 & \text{ml of 0.05 mol/L}\;{\text{KMn0}}_{4}\\ & =2\text{mg sodium oxalate equivalent/g of sample}. \end{array}$$

#### 2.6.3 Determination of saponin

The spectrophotometric method of Brunner (1984) was used for saponin analysis. One gram of finely ground sample was weighed into 250‐ml beaker and 100 ml of isobutyl alcohol was added. The mixture was shaken for 2 hr to ensure uniform mixing. Thereafter, the mixture was filtered through a Whatman No. 1 filter paper into a 100‐ml beaker and 20 ml of 40% saturated solution of magnesium carbonate was added and the mixture made up to 250 ml. The mixture obtained with saturated MgCO_3_ was again filtered through a Whatman No. 1 filter paper to obtain a clear colorless solution. One milliliter of the colorless solution was pipetted into a 50 ml volumetric flask and 2 ml of 5% FeCl_3_ solution was added and made up to mark with distilled water. It was allowed to stand for 30 min for blood red color to develop. Saponin stock solution was prepared and 1–10 ppm standard saponin solutions were prepared from saponin stock solution. The standard solution was treated similarly with 5% of FeCl_3_ solution as done for 1 ml of sample above. A dilution of 1–10 was made from the prepared solution. The absorbances of the samples as well as that of the standard solution were read after color development in a 752S Spectrum lab UV, VIS Spectrophotometer at a wavelength of 380 nm.

$$\begin{array}{cc} \text{Saponins}=\\ & \frac{\text{Absorbance of sample}\times \text{dil.factor}\times \text{gradient of standard graph}}{\text{sample weight}\times 10,000}\left(\text{mg/g}\right) \end{array}$$

### 2.7 Effect of pH and NaCl concentration on emulsifying activity index and emulsion stability index

The effect of pH on emulsifying activity index (EAI) and emulsion stability was determined by the method described by Pearce and Kinsella (1978) with slight modifications. About 500 mg of the samples was dispersed in 100 ml of distilled water at different NaCl concentrations (0.0, 0.5 and 1.0 mol/L). The pH of the protein solution was then adjusted separately to pH 2–10 with either 1 N HCl or 1 N NaOH and the solution was mixed with 50 ml of pure Gino oil and homogenized using a blender (SN2200 Qlink, Beijing, China) set at high speed for 60 s. Approximately, 50 ml aliquot of the emulsion was transferred from the bottom of the blender after homogenization and mixed with 5 ml of 0.1% sodium dodecyl sulfate solution. The absorbance of the diluted solution was then measured at 500 nm using spectrophotometer (722‐2000 Spectronic 20D, England).

The absorbance obtained was used to calculate the EAI as shown below;

$$\text{Emulsifying activity index}\left(m^{2}/g\right)=\frac{2\times 2.303\times A}{0.25\times \text{protein weight (g)}}$$

where, *A* is absorbance at 0 min after homogenization.

To determine the emulsion stability, the emulsions were allowed to stand for 10 min at room temperature and the ESI was determined as described below and expressed based on the absorbance at 0, 10 min, and the time difference as shown in the formula;

$$\text{Emulsion stability index}\left(\%\right)=\frac{\text{EAl at}0\text{min}}{\text{EA1 at}10\text{min}}\times 100$$

### 2.8 Effect of pH and NaCl concentration on foaming capacity and stability

A modified method described by Chavan et al. (2001) was used for the determination of the foaming capacity (FC) and foaming stability (FS) of the samples as influenced by salt concentration and pH. To 100 ml of NaCl solution at concentrations of 0.0, 0.5, and 1.0 was added 2 g of the samples. The pH of the protein solution was separately modified to pH 2, 4, 6, 8, and 10 with either 1 mol/L NaOH or 1 mol/L HCl until the set pH was achieved. The resultant solution was standardized for 2 min using a blender (SN2200 Qlink, Beijing, China) set at high speed. This was then transferred into a 250 ml measuring cylinder and volume increase to the original volume of solution in measuring cylinder noted. Percentage volume increase to that of the original volume of protein solution in the measuring cylinder was calculated and expressed as foaming capacity or whippability. Foam stability was expressed as a percentage of the volume of foam remaining in the measuring cylinder to that of the original volume after 30 min of quiescent period.

#### 2.8.1 Statistical analysis

All the analyses were conducted in triplicate and subjected to statistical analysis using analysis of variance (ANOVA). Means were separated using Duncan's multiple range test.

## 3 RESULTS AND DISCUSSION

### 3.1 Chemical composition of cooked and fermented kariya seeds

Table 1 shows the chemical composition of the fermented and cooked kariya seed. Moisture content was observed to range between 9.67% and 16.52% with cooked unfermented kariya (CUK) flour having the lowest moisture content and cooked fermented kariya (CFK) exhibiting the highest moisture content. The moisture content was observed to increase with fermentation days. The moisture content achieved in this study were within the range of values stated for pigeon pea flour (10.20%–13.24%) and tiger nut flour samples (9.93%–10.42%) by Adebowale and Maliki (2011) and Adejuyitan, Otunla, Akande, Bolarinwa, and Oladokun(2009) but higher than the values reported by (Osungbade, Olasunkanmi, & Oladipupo, 2016) for sandbox (*Hura crepitans*) seeds, respectively.

**Table 1:** Proximate composition of kariya flour

Sample	Moisture (%)	Protein (%)	Crude fats (%)	Ash (%)	Crude fiber (%)	Carbohydrate (%)
RFK	13.34 ± 0.00^c^	42.44 ± 0.00^b^	9.76 ± 0.09^c^	7.58 ± 0.39^abc^	4.90 ± 0.89^b^	36.92 ± 027^b^
RUK	10.92 ± 0.26^ab^	33.91 ± 1.99^a^	9.21 ± 0.03^b^	6.53 ± 0.04^ab^	1.68 ± 0.15^a^	48.67 ± 2.22^d^
CUK	9.67 ± 0.47^a^	33.93 ± 0.18^a^	9.00 ± 0.06^b^	5.51 ± 0.06^a^	1.71 ± 0.54^a^	49.84 ± 0.34^d^
CFK	16.52 ± 0.03^d^	44.57 ± 2.21^b^	8.36 ± 0.22^a^	8.25 ± 0.27^bc^	3.11 ± 1.53^ab^	35.89 ± 0.47^a^
CFK 1	9.91 ± 0.18^a^	34.99 ± 1.30^a^	8.36 ± 0.14^a^	6.74 ± 0.03^abc^	4.27 ± 0.54^b^	45.64 ± 1.96^d^
CFK2	11.56 ± 0.69^b^	36.64 ± 1.69^a^	8.39 ± 0.25^a^	8.61 ± 2.29^bc^	3.56 ± 0.21^b^	42.79 ± 0.56^c^
CFK3	14.06 ± 0.03^c^	42.79 ± 0.02^b^	8.45 ± 0.08^a^	8.97 ± 0.06^c^	4.07 ± 0.33^b^	35.72 ± 0.38^b^

RFK, raw fermented kariya (96 hr); RUK, raw unfermented kariya; CUK, cooked unfermented kariya; CFK, cooked fermented kariya (96 hr); CFK 1, cooked fermented kariya (24 hr); CFK 2, cooked fermented kariya (48 hr); CFK 3, cooked fermented kariya (72 hr).

Values reported are means ± standard deviation of triplicate determinations. Mean values with different superscript within the same column are significantly (*p* < .05) different.

Increased duration of fermentation period was observed to increase (*p* ≤ .05) the crude protein content of kariya flour. The crude protein of kariya flour samples increased (*p* ≤ .05) with fermentation period. The protein content ranged between 33.91% and 44.57% with the raw unfermented sample (RUK) having the lowest protein value, while the cooked fermented kariya (CFK) had the highest protein content. The increase in protein during fermentation observed in this study agrees with previous values (21.8%–23.9% and 24.08%–52.10%) reported for pigeon pea (*Cajanus cajan*) and sand box seed flour (Adebowale & Maliki, 2011; Osungbade et al., 2016). The increase in protein confers a nutritional benefit on the fermented kariya seed flour. The increase in protein value with fermentation time observed in this study could be ascribed to the net synthesis of protein by fermenting seeds (Uwagbute, Iroegbu, & Eke, 2000). This agrees with other scientific findings that processing techniques such as fermentation improve the nutritional quality of the food products, particularly in terms of protein content (Enujiugha, 2003; Fasasi, Eleyinmi, & Oyarekua, 2009).

As shown in Table 1, crude fat content ranges between 8.16% and 9.12% and were significantly different (*p* ≤ .05) among nonfermented and fermented samples. However, a significant difference (*p* ≤ .05) between all fermented samples was not observed. Increased lipolytic enzyme activities during the fermentation process might be responsible for the decrease in fat content observed in these set of samples. Lipolytic enzymes are known to hydrolyze fat components into glycerol and fatty acid.

Ash and carbohydrate content ranged from 5.51% to 8.97% and 35.72% to 49.84%, respectively, with nonfermented seed flour CUK having the lowest value (5.51%), while the highest value (8.97%) was recorded for CFK3 at the end of 72 hr fermentation for the ash content. CFK3 had the lowest carbohydrate value (35.72%), while the highest carbohydrate value (49.84%) was recorded for CUK. Ash contents increase with fermentation days, while carbohydrate value was observed to decrease with fermentation days. Observed changes in carbohydrate with fermentation time validates earlier reports by Onweluzo and Nwabugwu (2009), Nnam (1995), Achinewhu and Isichei (1990) who made similar findings on fermented pumpkin seeds, fermented cowpea, and fermented millet, respectively.

### 3.2 Effect of cooking and fermentation on oil absorption capacity

The effects of fermentation on the functional properties of kariya seeds flour are shown in Table 2. The oil absorption capacity (OAC) values range between 47.73% and 84.50% with sample CUK having the highest and RFK having the lowest. OAC values between samples, CUK and RUK were significantly different (*p* < .05) in contrast to samples RFK and CFK, where there was no significant difference (*p* < .05) between samples. Fermentation decreased the oil absorption capacity in both raw {(from 62.47% (RUK) to 47.73% (RFK)} and cooked {(84.50% (CUK)} to 51.63% (CFK)} flour samples. This observation shows a divergence from an earlier report by Adejuyitan et al. (2009) on tiger nut flour where OAC decreases with fermentation time. Also, a marginal increase in OAC with fermentation time on pea and *artocarpus altilis* have been earlier reported by Periago, Vidal, and Ros (1998) and Appiah, Oduro, and Ellis (2011). Low‐density protein powders with a small particle size have been reported to absorb and ⁄ or entrap more oil than do high‐density protein powders (Gbadamosi et al., 2012; Kadiri, Akanbi, Olawoye, & Gbadamosi, 2016). Low oil absorption capacity exhibited by samples RFK and CFK may be related to its high density and large particle size as proposed by Gbadamosi et al. (2012).

**Table 2:** Functional properties of kariya seed defatted flours at their natural pH

Functional characteristics	RUK	RFK	CUK	CFK
OAC (%)	62.47 ± 0.00^b^	47.73 ± 0.04^a^	84.50 ± 0.03^c^	51.63 ± 0.07^a^
EAI (m^2^/g)	8.75 ± 0.03^a^	29.79 ± 0.03^c^	24.19 ± 0.02^b^	24.95 ± 0.02^b^
ESI (%)	59.85 ± 0.03^a^	109.78 ± 0.03^c^	167.43 ± 0.03^d^	84.69 ± 0.03^b^
FC (%)	33.33 ± 0.03^d^	26.32 ± 0.03^c^	20.00 ± 0.17^b^	14.29 ± 0.03^a^
FS (%)	14.29 ± 0.03^b^	15.79 ± 0.03^d^	15.00 ± 0.03^c^	9.52 ± 0.03^a^
IVD (%)	63.71 ± 0.02^a^	82.14 ± 0.01^c^	65.50 ± 0.02^ab^	85.50 ± 0.04^d^

RUK, raw unfermented kariya; RFK, raw fermented kariya (96 hr); CUK, cooked unfermented kariya; CFK, cooked fermented kariya (96 hr).

Values reported are means ± standard deviation of triplicate determinations. Mean values with different superscript within the same row are significantly (*p* < .05) different.

### 3.3 Effect of cooking and fermentation on protein digestibility (IVPD)

Protein digestibility of all samples is shown in Table 2. Cooking and fermentation causes significant (*p* ≤ .05) improvement in IVPD in all samples. The increase was in the range of 63.71% to 85.51% in all samples (RFK, CUK, RUK, CFK, CFK1, CFK2, and CFK3). The cooking process increased the rate at which the antinutrients were leached out and destruction of protease inhibitors. The improvement in protein digestibility caused by fermentation could be attributed to partial degradation of complex proteins to more simple and stable products which could be a possible reason for the improvement of the protein digestibility of fermented samples. Hassan and El Tinay (1995) attributes this effect to the degradation of polyphenols, tannins, and phytic acid by microbial enzymes. Also, elimination of phytic acid during the degradation process by microbial enzymes during fermentation results in the improvement of protein digestibility in fermented millet (Khetarpaul, 1991).

### 3.4 Effect of temperature on water absorption capacity and swelling capacity

**Figure 1:**
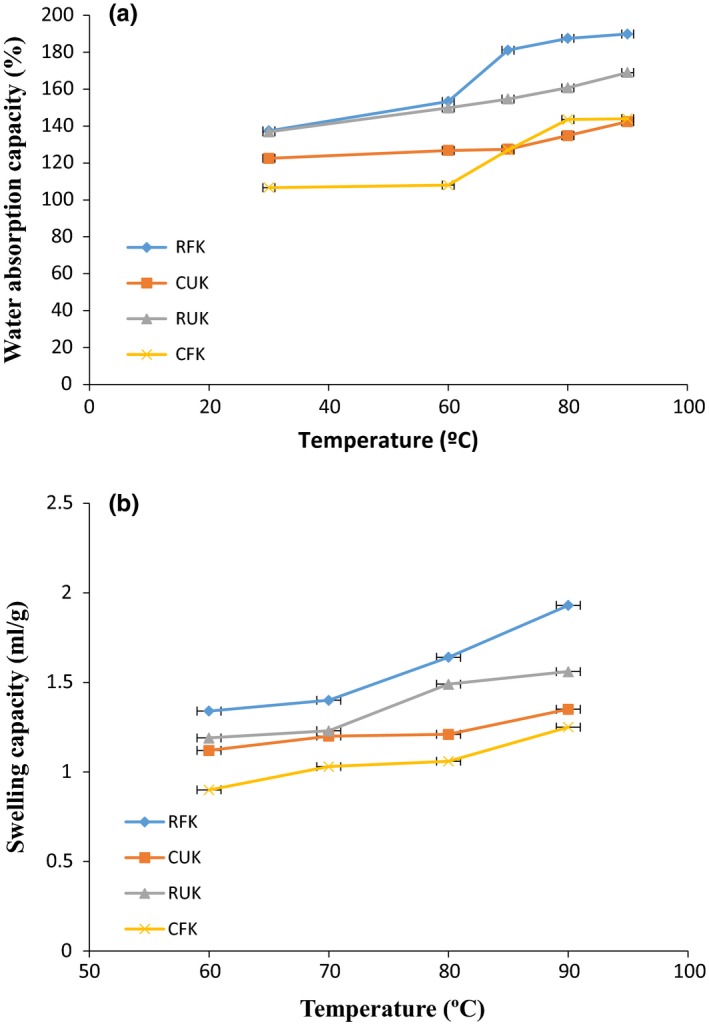
Effect of temperature on (a) water absorption capacity (%) of RFK, raw fermented kariya; CUK, cooked unfermented kariya; RUK, raw unfermented kariya; CFK, cooked fermented kariya (b) swelling capacity of RFK, CUK, RUK, CFK

The result of swelling capacity as influenced by temperature is shown in Figure 1. The swelling capacity values of RFK, CUK, RUK, and CFK between 30°C and 90°C ranged between 1.34 and 1.93 ml/g, 1.12 and 1.35 ml/g, 1.19 and 1.56 ml/g, and 0.90 and 1.25 ml/g, respectively, with lowest swelling capacity occurring at 60°C and highest at 90°C. The swelling capacity was observed to increase with an increase in temperature of all samples (Figure 1b). Swelling capacity is a function of processing conditions, material nature, and treatment type. Biopolymers such as starch are involved in the improvement of these characteristics. The study revealed a decrease in swelling capacity of the cooked kariya flour compared to the native or raw kariya flour. This might be due to pregelatinization and predenaturation of starch and protein, respectively, during the cooking process. The high swelling power achieved at 80°C and 90°C suggest that water penetration into the granules can be achieved at elevated temperatures and this could be useful in the manufacture of confectionary foods (that requires the high swelling power of starch). The swelling capacity obtained in this result falls within the range of values reported for *Artocarpus artilis* flour, 0.95 (60°C) to 3.76 (90°C) as reported by Adepeju et al. (2011).

The result of the effect of temperature on water absorption capacity of defatted kariya seed flour is shown in Figure 1a. The WAC of the fermented and unfermented kariya flour was observed to increase with temperature increase (30°C–90°C). The WAC values of RFK, CUK, RUK, and CFK ranged between 137.47% and 189.90%, 122.50% and 142.37%, 136.90% and 169.03%, and 106.63% and 144.00%, respectively, with the highest amount being absorbed at 90°C. At higher temperatures (80°C and 90°C), there was no significant difference in the WAC of CUK and CFK but the values were significantly lower than those of RFK and RUK. The increase in water absorption capacity of flour with increasing temperature is in agreement with earlier reports by Osundahunsi, Fagbemi, Kesselman, and Shimoni (2003) in their study on the physicochemical properties and pasting characteristics of flour and starch from red and white sweet potato cultivars. Likewise, Fasasi et al. (2009) reported exhibited increased WAC with increasing temperature on fermented maize flour. Defatted kariya flour from this study could be used as thickeners in either liquid or semi‐solid foods due to its high WAC properties at high temperatures recorded.

### 3.5 Effect of pH and salt (NaCl) concentrations on emulsifying activity index (EAI) and emulsion stability index (ESI) of cooked and fermented kariya seeds

**Figure 2:**
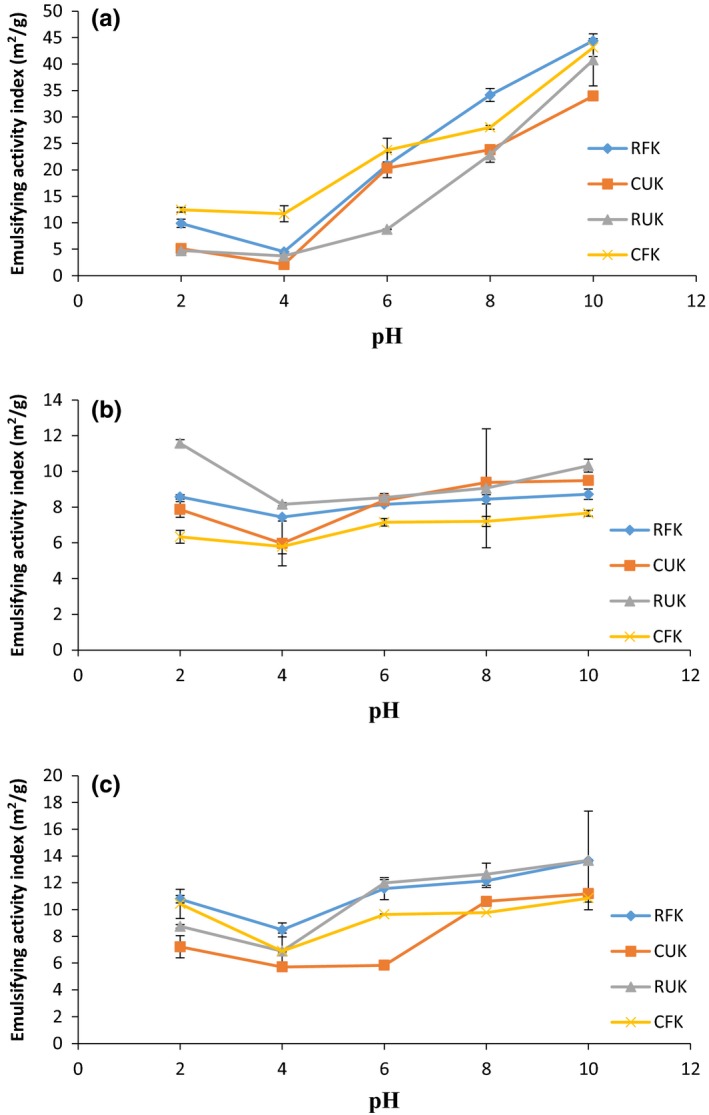
Emulsion activity index (m^2^/g) of RFK, CUK, RUK, and CFK at (a) 0.0 mol/L, (b) 0.5 mol/L, and (c) 1.0 mol/L NaCl concentration as a function of PH

**Figure 3:**
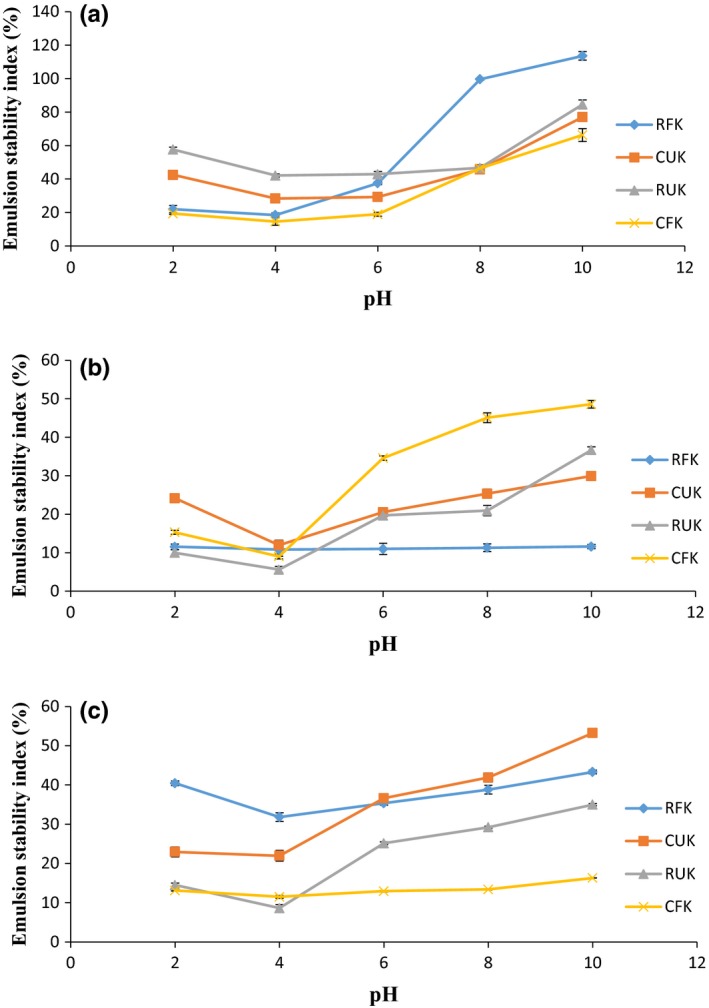
Emulsion stability index (%) of RFK, CUK, RUK, and CFK at (a) 0.0 mol/L, (b) 0.5 mol/L, and (c) 1.0 mol/L NaCl concentration as a function of PH

**Figure 4:**
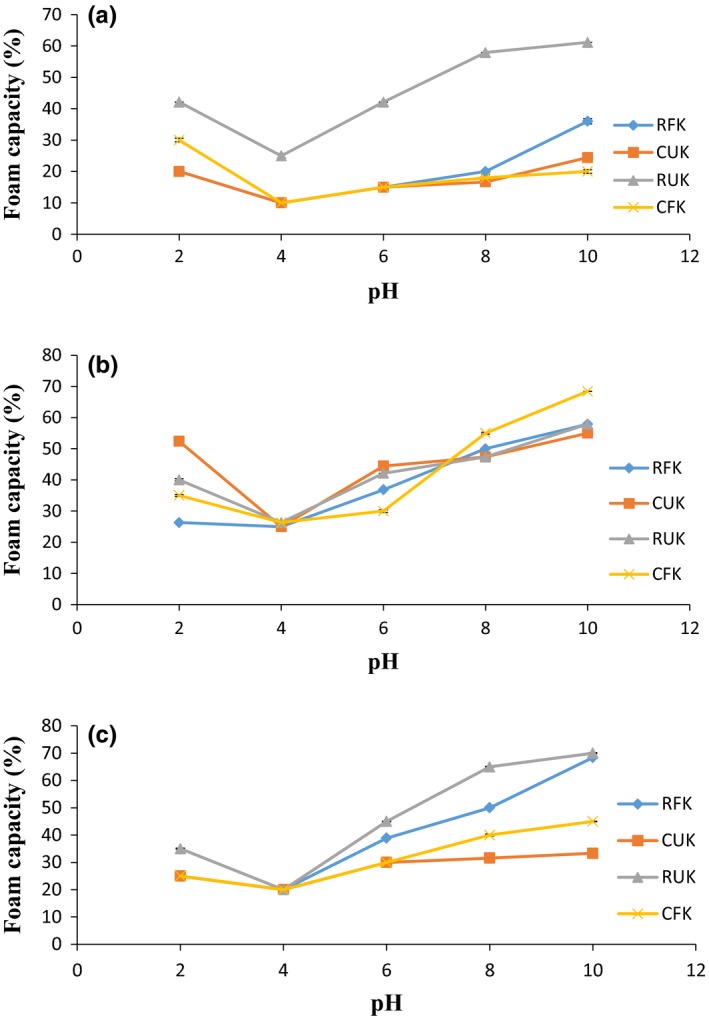
Foam capacity (%) of RFK, CUK, RUK, and CFK at (a) 0.0 mol/L, (b) 0.5 mol/L, and (c) 1.0 mol/L NaCl concentration as a function of PH

**Figure 5:**
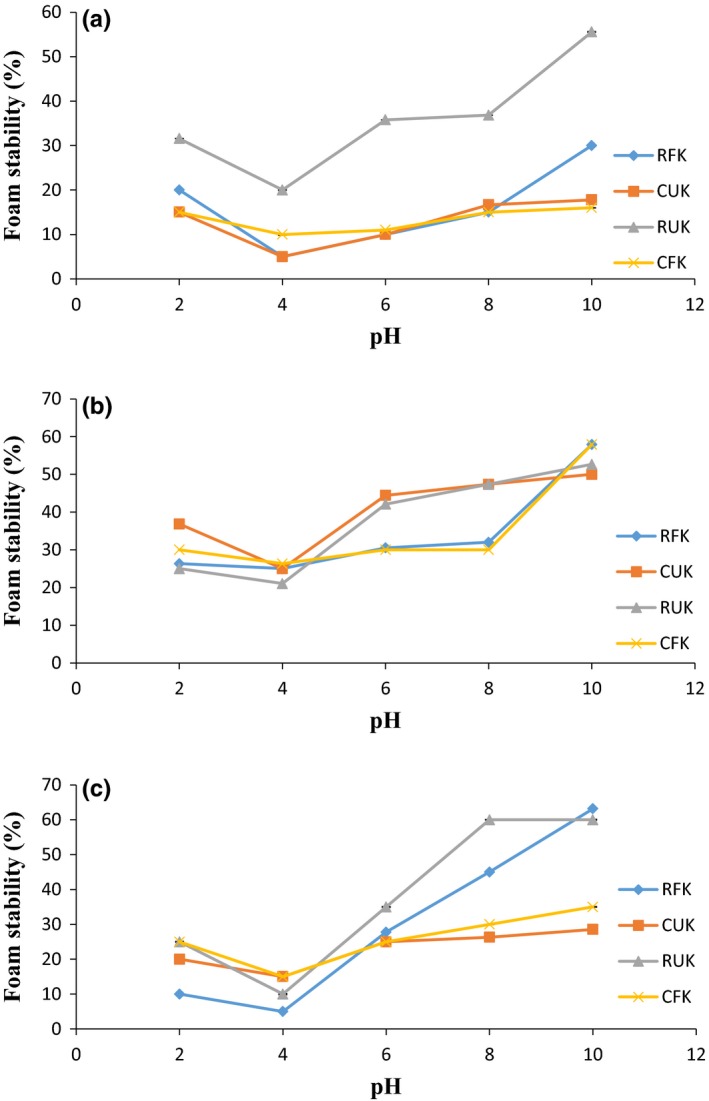
Foam stability (%) of RFK, CUK, RUK, and CFK at (a) 0.0 mol/L, (b) 0.5 mol/L, and (c) 1.0 mol/L NaCl concentration as a function of PH

The EAI and ESI at neutral pH of the samples ranged between 8.75 m^2^/g and 29.79 m^2^/g and 59.85% and 167.43%, respectively (Table 2). Raw unfermented Kariya seed (RUK) had the lowest value for EAI and ESI (8.75 m^2^/g and 59.85%, respectively) which was significantly different (*p* < .05) from the other samples; whereas raw fermented Kariya (RFK) had the highest EAI (29.79 m^2^/g) and cooked unfermented Kariya (CUK) had the highest ESI (167.43%). The high EAI value for RFK and as seen in CUK and CFK might be due to protein denaturation caused by the fermentation and the cooking process which exposed more hydrophobic groups. Denaturation has been revealed to increase the emulsifying properties of proteins, due to improved hydrophobic surface and flexibility (Dickinson & Hong, 1994; Moure, Sineirob, Domíngueza, & Parajó, 2006). The values attained in this work are within the range of values obtained for conophor defatted flour (CDF), conophor protein concentrate (CPC), isoelectric protein isolate (CII), and neutral protein isolate (CNI) (40.70, 27.15, 24.42, 20.17 m^2^/g) as reported by Gbadamosi et al. (2012). Raw, cooked, or fermented kariya seed flours could be use as food ingredients in food formulation and as additive for the stabilization of emulsions in soups and cakes based on the result of the findings discussed previously. The lowest EAI and ESI of RFK (4.51 m^2^/g and 18.49%), CUK (2.12 m^2^/g and 28.33%), RUK (3.74 m^2^/g and 42.05%), and CFK (11.68 m^2^/g and 14.57%), respectively, were establish around the isoelectric pH established earlier, while the highest EAI and ESI of RFK (44.40 m^2^/g and 113.59%), CUK (33.97 m^2^/g and 76.98%), RUK (40.77 m^2^/g and 84.57%), and CFK (43.13 m^2^/g and 66.25%), respectively were observed at pH 10 and the differences were significant (*p* < .05) as shown in Figure 2. According to Adiamo et al. (2015a, 2015b), most food proteins are poorly hydrated, lack electrostatic repulsive forces, sparingly soluble, and are generally poor emulsifiers at the isoelectric pH. The EAI of the samples was observed to increase after the isoelectric pH point with increased salt concentration when salt concentration was increased from 0.5 to 1.0 mol/L. At high salt concentrations, salts compete with protein for water resulting in low extractability of protein due to the salting out effect at this concentration (Osungbade et al., 2016) which is a possible explanation for the decrease in EAI of samples under study. Salting out occurs at high salt concentrations when salts compete with the protein for water (Adiamo et al., 2015a, 2015b) (Figures 3, 4, 5).

At 0.5 mol/L NaCl, there was a reduction in EAI and ESI values which showed a minimal increase as the salt concentration was increased to 1.0 mol/L. This observation agrees with an earlier report by Osman, Amro, Mohamed‐Ahmed, and Babiker (2005) in which it was observed that at 0.0 mol/L NaCl, the EAI and ESI of chickpea were higher but afterward decreased at 0.2 mol/L NaCl although no marginal reduction occurred at 0.6 mol/L NaCl. This trend has been affirmed by previous research on the effect of salt concentration on conophor proteins in a study by Gbadamosi et al. (2012). This investigator reported that at high salt concentration, EAI values were low and attributed this to the increase in repulsive forces which is as a result of the poor interfacial properties of the interfacial film of protein molecules. At high salt concentration, negatively charged chloride ions are induced to interact with positively charged proteins, thereby resulting in a reduction in electrostatic repulsion and enhance hydrophobic interaction. This results in a higher tendency for the protein to form insoluble aggregates which result in decreasing solubility and emulsifying activity. This observation by this investigator is also in agreement with the previous report on pea and hyacinths bean as reported by Ahmed, Babiker, Mohamed Ahmed, Eltayeb, and Faridullah (2012).

### 3.6 Effect of pH and salt (NaCl) concentrations on the foaming capacity and stability of cooked and fermented kariya seeds

The FC and FS at neutral pH of the kariya flour samples ranged between 14.29% and 33.33% and 9.52% and 15.79%, respectively (Table 2). The FC of RUK (33.33%) was higher than those of RFK (26.32%), CUK (20.00%), and CFK (14.29%), and significantly different (*p* < .05) from one another. Raw unfermented Kariya seed flour (RUK) had better foaming capacity which could be due to improved solubility and swift unfolding of its protein at the air‐water interface. The FC of RUK is similar to those of conophor defatted flour (35%) and pra seed flour (32.6%) reported by Gbadamosi et al. (2012) and Choonhahirun (2010). The lower FC and FS as observed in CUK and CFK (cooked samples) as compared to raw samples might be as a result of the heating process (cooking) that may have denatured the protein and reduced its solubility. The result obtained in this study is in agreement with the report of Nwosu (2010) in which the foaming capacity and stability of oze seed flour were significantly reduced when subjected to heat.

Figure 4 and 5 shows the effect of pH and NaCl at 0.0 mol/L, 0.5 mol/L, and 1.0 mol/L on the foaming capacity (FC) and stability (FS) of the various samples. The lowest FC and FS for RFK (10.00% and 5.00%), CUK (10.00% and 5.00%), RUK (25.00% and 20.00%), and CFK (10.00% and 10.00%), respectively, were observed at 0.0 mol/L NaCl and pH 4, which was also the pH at which these protein exhibit the lowest emulsifying and solubility properties. FC and FS were highest at pH 10 for RFK (36% and 30.00%), CUK (24.44% and 17.78%), RUK (61.11% and 55.56%), and CFK (20.00% and 16.00%), respectively. Foaming properties of RUK (at 0.0 mol/L NaCl) was higher than those of the other samples (RFK, CUK, and CFK) at all pH values (2–10) and significant (*p* ≤ .05). The presence of the native protein in RUK compared to denatured proteins in RFK, CUK, and CFK are likely to reason for this observation.

Increase in foam capacity of the samples as salt concentration increases may be due to the fact that salt diminishes protein films rigidity surface viscosity while causing an increase in their spreading rate, thereby resulting in the weakening interpeptide interactions and consequently increase foam volumes for certain proteins (Oshodi, 1992). The result obtained in this study compared favorably with the reports of Berhanu and Amare (2013), on defatted soy bean which was found to exhibit increased foam capacity as the salt concentration increased. The foam stability of the samples was also found to increase as the salt concentration increases. This result obtained is in line with what was reported by Osman et al. (2005) and by Mahajan, Bhardwaj, and Dua (1999) who found out that as the concentration of NaCl increased, the foam stability of chickpea flour also increased significantly. From this report, it can be deduced that kariya seed flour foaming capacity and stability improves with increase in salt concentration. Arogundade, Akinfenwa, and Salawu (2004)) previously reported that improved foaming capacity and stability of seed flour in the presence of NaCl will improve its functional properties and invariably its application in food processing such as frosting, whipped toppings, and cake mixes where foaming is of vital prominence.

### 3.7 Effect of cooking and fermentation on the antinutritional content of kariya flour

The effects of cooking and fermentation on the antinutrient levels of kariya flour samples are shown in Table 3. All the flour samples showed a reduction in oxalate (8.13–6.50* *mg/100 g), tannins (1.63–1.20 mg/100 g), and saponins (0.18–013 mg/100 g), respectively. The values of antinutrient of all samples with respect to the tested antinutrient were significant differences (*p* < .05). The result showed that the concentrations of each tested antinutrient reduced significantly (*p < *.05) with an increase in the duration of fermentation days. The result obtained in this study compares favorably with the work of Fowomola and Akindahunsi (2008) who reported that an increase in fermentation days caused a significant reduction in the antinutrient (oxalate, tannin, saponin, alkaloid, phytate, and cyanide) contents present in *Hura crepitans* seed. The observed progressive reductions of antinutritional factors as the fermentation progresses is in line with the assertion that the antinutrient levels were generally reduced due to the microflora activities and secreted polyphenol oxidase (Achinewhu & Isichei, 1990). The activities of polyphenol oxidase secreted by fermentative micro flora on tannin might have caused a reduction in tannin content during the processing of the seed flour (Reddy & Pierson, 1994).

**Table 3:** Antinutritional properties of cooked and fermented flour (RFK, CUK, RUK, and CFK)

Sample	Tanin (mg/100 g)	Saponin (mg/100 g)	Oxalate (mg/100 g)
RFK	1.47 ± 0.01^d^	0.13 ± 0.01^a^	7.26 ± 0.01^c^
CUK	1.54 ± 0.01^e^	0.16 ± 0.01^c^	7.79 ± 0.01^e^
RUK	1.63 ± 0.01^f^	0.18 ± 0.01^d^	8.13 ± 0.01^f^
CFK	1.20 ± 0.01^a^	0.13 ± 0.01^a^	6.50 ± 0.01^a^
CFK 1	1.44 ± 0.02^d^	0.15 ± 0.01^bc^	6.93 ± 0.01^b^
CFK2	1.27 ± 0.01^b^	0.14 ± 0.01^ab^	6.56 ± 0.04^a^
CFK3	1.23 ± 0.01^a^	0.13 ± 0.00^ab^	6.53 ± 0.03^a^

RFK, raw fermented kariya (96 hr); CUK, cooked unfermented kariya; RUK, raw unfermented kariya; CFK, cooked fermented kariya (96 hr); CFK 1, cooked fermented kariya (24 hr); CFK 2, Cooked fermented kariya (48 hr); CFK 3, cooked fermented kariya (72 hr).

Values reported are means ± standard deviation of triplicate determinations. Mean values with different superscript within the same column are significantly (*p* < .05) different.

Also, the cooking process as observed in samples CFK, CFK1, CFK2, and CFK3 resulted in significant reduction in the antinutrients of samples coupled with the fermentation process. Provided that food is adequately cooked to certain temperature and time duration, the toxic effect of antinutrients such as tannin, oxalate, or phytate will be significantly reduced to a permissible level which is nonharmful to health (Enechi & Odonwodu, 2003). Similar observation was reported by Ikemefuna, Obizoba, and Atii (1991) where the combination of cooking and fermentation was reported to drastically lessen the antinutritional factors in sorghum (*Guinesia*) seeds drastically to a safe level. The values of the antinutrients observed in this study were lesser than the values (tannin: 18.61–5.8 mg/100 g; oxalates: 23.7–3.6 mg/100 g; and saponins: 8.5–1.4 mg/100 g) reported by Fowomola and Akindahunsi (2008) that cooking coupled with increase in fermentation days result in significant reduction in the antinutrients content of *Hura crepitans* seed. The results agreed with the reports of Ibukun and Anyasi (2012) where a reduction was observed in the oxalate, tannin, and saponin levels of fermented sesame seeds (*Sesanum indicum*) (2.57–0.36, 0.019–0.008, and 2.68–1.01 mg/g extract, respectively); musk melon seeds (*Cucumis melo*) (2.1–0.27, 0.007–0.004, and 5.1–2.8 mg/g extract, respectively) and; white melon seeds (*Cucumeropsis mannii*) (1.35–0.14, 0.008–0.005, and 3.5–1.9 mg/g extract), respectively. Therefore, a combination of cooking and fermentation will result in significant reduction in the antinutrients and improvement of the nutritional quality of kariya seeds.

## 4 CONCLUSION

Cooking and fermentation of *H*. *barteri* seeds significantly enhance their protein contents and digestibility. The antinutritional content was also observed to be decreased to safe and acceptable levels after subjecting to fermentation and cooking at conditions stated in this study. Likewise, the processing method employed in this study improves the functional properties of kariya seed flour and is readily available for use as food ingredient in food formulation and supplementation.

## CONFLICT OF INTEREST

None declared.
